# MK2-mediated AKT/MYC signaling activation promotes epithelial-mesenchymal transition in lung adenocarcinoma

**DOI:** 10.3389/fgene.2025.1615018

**Published:** 2025-09-25

**Authors:** Rong Qi, Chen Fang, Penghui Liu, Weiguo Gu, Chao Shi, Guohua Zhang, Feng Qiu

**Affiliations:** ^1^ Department of Oncology, Gaoxin Branch of the First Affiliated Hospital of Nanchang University, Nanchang, China; ^2^ Nanchang Key Laboratory of Tumor Gene Diagnosis and Innovative Treatment Research, Gaoxin Branch of the First Affiliated Hospital of Nanchang University, Nanchang, China; ^3^ Department of Medical Oncology, The First Affiliated Hospital of Nanchang University, Nanchang, China

**Keywords:** lung adenocarcinoma, MK2, EMT, AKT/MYC pathway, CancerProgression

## Abstract

**Purpose:**

The protein kinase Mitogen-Activated Protein Kinase-Activated Protein Kinase 2 (MK2) is linked to higher risks of metastasis and mortality in some cancers. Nonetheless, its precise function in lung adenocarcinoma (LUAD) remains unclear. This study aims to explore MK2’s function in LUAD cells and identify the underlying molecular mechanisms.

**Methods:**

MK2 expression in LUAD patients was confirmed through Timer2.0 database and tissue microarrays. Immunohistochemical staining for MK2 was performed on LUAD samples to investigate its association with metastasis and invasion. The activity of MK2 was inhibited in LUAD cell lines A549 and H358 using a specific MK2 inhibitor. Subsequently, cell viability, migration, and invasion were assessed. Gene expression changes were confirmed through Western blotting. Additionally, an AKT activator was used to validate the role of the MK2-regulated AKT/MYC signaling pathway.

**Results:**

MK2 expression is significantly higher in LUAD tissues compared to adjacent normal tissues. Reducing MK2 activity not only curtails cell proliferation, migration, and EMT-related invasion *in vitro* but also disrupts the AKT/MYC signaling axis. Activation of the AKT/MYC pathway can counteract the inhibitory effects of MK2 suppression.

**Conclusion:**

Our findings suggest that MK2 promotes migration and invasion in LUAD through the AKT/MYC signaling pathways, positioning MK2 as a potential therapeutic target in LUAD treatment.

## 1 Introduction

Lung cancer is the leading cause of cancer-related morbidity and mortality worldwide (Sung et al., 2021). Within this category, non-small cell lung cancer (NSCLC) comprises 85% of all lung cancer cases, standing as the predominant form (Miller et al., 2022). Among NSCLC cases, lung adenocarcinoma (LUAD) emerges as the primary histological variant, representing approximately 40% of all malignant lung tumors (Chen et al., 2014). The clinical management of LUAD faces significant challenges due to frequent late-stage diagnosis, which correlates with unfavorable prognosis and elevated mortality rates. The epithelial-mesenchymal transition (EMT), a fundamental biological process characterized by the transformation of polarized epithelial cells into motile mesenchymal phenotypes, has been extensively implicated in tumor progression and metastatic dissemination (Behrooz et al., 2024; Coelho-Rato et al., 2024). Emerging evidence demonstrates that EMT activation not only facilitates tumor cell migration and tissue invasion but also critically influences cancer mortality through its role in therapeutic resistance and distant metastasis formation (Nie et al., 2025; Langley and Fidler, 2007). While molecular targeted therapies have revolutionized NSCLC treatment, substantial clinical limitations persist. Approximately 60%–70% of LUAD patients exhibit either non-targetable genetic profiles or develop acquired resistance to existing therapies (Hirsch et al., 2017; Le et al., 2018). This therapeutic impasse underscores the urgent need to elucidate the molecular underpinnings of LUAD metastasis, particularly EMT-mediated pathways. Our study focuses on identifying novel therapeutic targets within EMT regulatory networks, aiming to develop precision interventions that may simultaneously inhibit metastatic progression and improve long-term survival outcomes in LUAD patients.

Mitogen-activated protein kinase-activated protein kinase 2 (MK2), a serine/threonine protein kinase, acts as a downstream component of the p38 MAPK signaling pathway. This pathway is activated by environmental stress and plays a crucial role in promoting cell migration, motility, and metastasis. MK2-mediated phosphorylation events have been shown to orchestrate tumor invasiveness, with ​recent studies demonstrating that SUMOylation-dependent MK2/p38α interactions drive metastatic progression in gastric adenocarcinoma models (Wang et al., 2021). Clinical translational studies reveal the therapeutic implications of this pathway: GAGE7B-induced activation of the pMAPKAPK2/pHSP27 axis correlates with advanced disease progression and reduced 5-year survival rates in gastrointestinal malignancies (Henriques et al., 2018; Shi et al., 2019). Preclinical validation further supports MK2’s oncogenic role, as shRNA-mediated MK2 silencing suppresses multiple myeloma cell proliferation and induces caspase-3-dependent apoptosist (Gu et al., 2021). However, the role of MK2 in LUAD cell migration, motility, and proliferation remains unclear, and further investigation is needed.

The PI3K/AKT/mTOR signaling axis is a central driver of oncogenesis, exhibiting frequent activation across diverse malignancies through genetic alterations such as PTEN loss, AKT amplification, and receptor tyrosine kinase hyperactivation (Yu et al., 2022). This pathway critically sustains tumor cell proliferation and survival by dual regulatory mechanisms: suppressing pro-apoptotic factors through phosphorylation-dependent inactivation while concurrently enhancing anti-apoptotic effector expression (Tao et al., 2017). Beyond its role in cell survival, AKT signaling orchestrates metastatic progression by inducing EMT via transcriptional activation of key regulators including Twist1 and Snail, which collectively promote cadherin switching and extracellular matrix remodeling to facilitate tumor cell dissemination (Gao et al., 2024; Ma et al., 2024; Chen et al., 2021). The proto-oncogene MYC, overexpressed in many types of cancers, functions as a master coordinator of malignant transformation. MYC accelerates cell cycle progression through cyclin D/E upregulation and proliferation and invasion (Dang et al., 2006; Zhao et al., 2021; Zhu et al., 2022; Meskyte et al., 2020). Emerging evidence underscores intricate crosstalk between AKT and MYC, wherein AKT activates MYC through a variety of downstream mechanisms, such as directly or indirectly promoting MYC transcription and translation through the PI3K/AKT/mTOR pathway (Cao et al., 2022; Cai et al., 2021; Xu et al., 2019). Clinically, this AKT/MYC signaling nexus has been implicated in aggressive metastatic behaviors across multiple cancer types, including bladder carcinoma, nasopharyngeal cancer, lung cancer and breast cancer, where its activation correlates with advanced disease stages and reduced survival outcomes (Wei et al., 2019; Su et al., 2024; Sun et al., 2023b). Recent preclinical studies have indicated that the MK2-regulated AKT/MYC signaling pathway enhances tumor metastasis (Deng et al., 2018). However, the functional role of MK2 within the molecular landscape of lung adenocarcinoma (LUAD) remains poorly characterized, particularly regarding its capacity to regulate metastatic processes through AKT/MYC pathway interactions. Our study therefore focuses on examining the impact of MK2 on LUAD cell migration and invasion, and aims to define the contribution of the AKT/MYC pathway in MK2-driven tumor metastasis.

## 2 Materials and methods

### 2.1 Public database analysis

The expression profile of the target molecule, MK2, in LUAD was derived from online analysis using immune infiltration data for various cancer types (pan-cancer) sourced from the TIMER 2.0 database [http://timer.compgenomics.org/].

Additionally, survival analysis was conducted using the Kaplan-Meier method [https://kmplot.com/analysis/] on patient data extracted from the Kaplan-Meier plotter database for LUAD. Patients were classified according to the expression level of MK2 in their tumors, with the median value using the median value as a threshold to separate those with high expression from those with low expression.

### 2.2 Tissue microarray and immunohistochemistry

Shanghai Zhuoli Biotechnology Co., Ltd. provided LUAD tissue chip (ZL-LugA961), comprising 48 pairs of tumor tissue and adjacent non-tumor tissue samples. However, after analysis, only 47 pairs had sufficient data for immunohistochemistry (IHC) analysis, as one pair failed to exhibit adequate staining or could not be scored. Therefore, the final number of analyzable samples was 47. Microarrays underwent pretreatment with bovine serum albumin (BSA, Elabscience Biotechnology Co.,Ltd., China, Cat. No. E-IR-R107, validated for IHC use) before being incubated overnight at 4 °C with MK2 antibody from Proteintech (Cat. No. 13949-1-AP, validated for IHC, diluted 1:200). The following day, further incubation was conducted with HRP-labeled secondary antibodies. Visualization was achieved using DAB (ZSGB-BIO, Cat. No. ZLI-9017), followed by hematoxylin counterstaining (Beyotime, Cat. No. C0107), and images were captured utilizing a microscope. Finally, Visiopharm software facilitated quantitative analysis of staining intensity. The HDAB-DAB filter was used to segment regions of interest (ROIs) based on staining intensity. Intensity categories are as follows: 0–75 (strong), 76–120 (moderate), 121–160 (weak), and 161–212 (negative). The staining area is measured in square micrometers (μm^2^). Staining intensity was measured using the H-Score (H-SCORE = ∑(pi × i)), calculated by summing the products of the percentages of positively stained cells at each intensity level and their respective intensity levels (H-SCORE = ∑ (pi × i)). Staining intensities are categorized as weak (1), medium (2), or strong (3).

### 2.3 Hematoxylin-eosin staining

Tissue samples were fixed in 10% formalin, dehydrated through graded ethanol, embedded in paraffin, and sectioned at 4 µm thickness. Sections were stained with hematoxylin and eosin (H&E) to evaluate histological morphology under a light microscope, facilitating the assessment of structural and pathological features.

### 2.4 Patient-derived lung adenocarcinoma organoid culture and identification

Clinical specimens were collected from LUAD patients, cryopreserved, and promptly processed for digestion. The primary tumor cells obtained were embedded in Matrigel (bioGenous Biotechnology Co. Ltd, China, Cat. No. M315066, validated for organoid culture) for three-dimensional culture, with media changes every 2–3 days. Once the organoids reached an appropriate size, they were passaged and collected. The organoids were then fixed in 4% paraformaldehyde, embedded in paraffin blocks, and prepared for subsequent sectioning and experimentation. Detailed culture steps were performed as previously described (Kim et al., 2019). All culture reagents were sourced from bioGenous Biotechnology Co., Ltd. (Suzhou, China), and are certified for clinical and research use with quality validation provided by the manufacturer.

### 2.5 Cell cultivation and handling

The H358 and A549 LUAD cell lines were procured from the Cell Bank of the Chinese Academy of Sciences (Shanghai, China). These cells were maintained in RPMI 1640 medium (Solarbio, China) supplemented with 10% FBS. Culturing was performed at 37 °C under a humidified atmosphere containing 5% CO2. To assess MK2’s function, cells in the inhibitor group were treated with a specific MK2 inhibitor (Huang et al., 2011; Zhang et al., 2021). The MK2 inhibitor used in this study, MK2-IN-1 (MCE, Cat.No. HY-12834; CAS No. 1314118–92–7; molecular formula: C_27_H_25_ClN_4_O_2_), is a potent, non-ATP-competitive inhibitor designed to achieve high selectivity for MK2. For all experiments, MK2-IN-1 was dissolved in PBS, and cells in the MK2 inhibitor treatment groups were exposed to 20 μM MK2-IN-1 for 24 h prior to subsequent assays. The control group received no treatment. Subsequently, cells pre-treated with the MK2 inhibitor for approximately 6 h were exposed to the AKT/MYC pathway activator SC79 (20 μg/mL) in follow-up experiments to research the function of the AKT/MYC signaling pathway.

### 2.6 Western blotting

Cell lysis solution was made using RIPA buffer (Beyotime, China, Cat. No. P0013B) supplemented with protease and phosphatase inhibitors (Beyotime, Cat. No. P1045 and P1081). Proteins were isolated using SDS-PAGE (8%–15%) and transferred onto PVDF membranes (Millipore, Cat. No. IPVH00010). These membranes were blocked with 5% skim milk for a minimum of 1 hour. Primary antibodies for MYC (Proteintech, Cat. No. 10828-1-AP, 1:1000), GAPDH (Proteintech, Cat. No. 60004-1-Ig, 1:50,000), AKT (Proteintech,Cat. No. 10176-2-AP, 1:2000), P-AKT (Ser-473) (HUABIO, Cat. No. ET1607-73, 1:1000), E-Cadherin (Proteintech, Cat. No. 60335-1-Ig, 1:2000), Vimentin (Proteintech, Cat. No. 60330-1-Ig, 1:20,000), N-Cadherin (Abcolonal, Cat. No. A0433, 1:500), and MMP2(Proteintech, Cat. No. 66366-1-Ig, 1:1000) were incubated at 4 °C overnight. After three washes with PBST, the HRP-labelled secondary antibody was incubated for 1 hour. After that, protein detection was performed using an ultra-high sensitivity ECL (GLPBIO, USA, Cat. No. GK10008).

### 2.7 RT-qPCR

Total RNA was extracted from cells using Eastep total RNA extraction kit (Promega, Cat. No. LS1040) following the manufacturer’s instructions, and RNA quality was assessed by spectrophotometry. cDNA synthesis was performed with Superscript III reverse transcriptase (Applied Biosystems) using 1 μg of RNA as the template, under conditions specified by the manufacturer. Real-time quantitative PCR (qPCR) was carried out using a PerfectStart^®^ Green qPCR Super Mix on a Roche with specific primers for target genes and we quantified GAPDH mRNA levels as an internal quantity control. Each reaction included 2 μL of cDNA template in a final reaction volume of 20 μL. Cycling conditions included an initial denaturation step, followed by 40 cycles of denaturation, annealing, and extension. Specificity was confirmed with melt curve analysis. Relative gene expression was calculated using the 2^(-^ΔΔ^Ct) method, normalized to GAPDH. Reactions were performed in triplicates, and results were analyzed using GraphPad Prism to determine statistical significance. All primers used for RT-qPCR were obtained from Sangon Biotech (Shanghai, China). RT-qPCR products were then subjected to electrophoresis. The primer sequences of target genes are listed in Table 1.

**TABLE 1:** Primer sequences for target genes.

Gene	Primer sequences (5′to 3′,forward to reverse)
MMP2	Forward:5′-CACCAAGAACTTCCGTCTGTCC-3′ Reverse:5′-GTGCCAAGGTCAATGTCAGGAG-3′
Vimentin	Forward:5′-GCAGGACTCGGTGGACTTCTC-3′ Reverse:5′-GTAGTTGGCGAAGCGGTCATTC-3′
E-Cadherin (CDH1)	Forward:5′-TCTGCTGCTCTTGCTGTTTCTTC-3′ Reverse:5′-TCTTCTCCGCCTCCTTCTTCATC-3′
N-Cadherin (CDH2)	Forward:5′-GACAGTTCCTGAGGGATCAAAGC-3′ Reverse:5′-TGGAGCCTGAGACACGATTCTG-3′
Snail2	Forward:5′-CCATGCCTGTCATACCACAACC-3′ Reverse:5′-TGGAATGGAGCAGCGGTAGTC-3′

### 2.8 Co-immunoprecipitation (CO-IP)

Cells were lysed in ice-cold IP lysis (Beyotime, China, Cat. No. P0013) buffer containing protease inhibitors. The lysates were incubated with specific antibodies against AKT (Proteintech, Cat. No. 10176-2-AP), followed by the addition of magnetic beads (Protein A or G Magnetic Beads, BeaverBio™). After incubation and washing, protein complexes were eluted and separated by SDS-PAGE. Western blotting with anti-MK2 and anti-AKT antibodies confirmed the interaction.

### 2.9 Annexin V-apc/7-AAD double staining

The organization referred to as “LUAD cells” typically denotes lung adenocarcinoma cells. They were cultured in six-well plates with a density of 100,000 cells per well for 24 h. Following a PBS wash, the cells received MK2 inhibitor treatment for an additional 24 h. Subsequently, the cells were promptly fixed and stained in accordance with the Annexin V-APC/7-AAD apoptosis kit protocol (Elabscience, Cat. No. E-CK-A218, validated for flow cytometry), with immediate flow cytometry analysis thereafter.

### 2.10 Cell migration assay

Wound healing assays were employed to evaluate cell migration ability. Cells were seeded in six-well plates at a density of 4 × 10^5^ cells per well and permitted to grow overnight to establish a monolayer. After making scratches using 200 µL pipette tips, they were washed with PBS and incubated in RPMI 1640 medium with 1% FBS for 24 h. Wound closure was measured using a Leica DMI8 fluorescence microscope at 0 and 24 h after wounding. All experiments were performed in triplicate.

### 2.11 Invasion assay

We utilized Matrigel matrix provided by BD Biosciences (BD Biosciences, USA) diluted at a ratio of 1:6 in serum-free RPMI 1640 medium, and stored it at 4 °C. After evenly spreading 100 μL of the matrix solution onto the surface of the upper chamber, we allowed it to dry for 1 h at 37 °C. Subsequently, cells were suspended in serum-free medium at a concentration of 5 × 10^5^cells/mL. Then, we added 200 μL of the cell suspension to the upper chamber coated with the matrix solution, while filling the lower chamber with RPMI 1640 medium containing 10% FBS. After 24 h, cells in the lower chamber were fixed with 4% paraformaldehyde for 30 min and then stained with 0.1% crystal violet for another 30 min. Following this, the stained cells were counted using a Leica fluorescence inverted microscope (DMI8). Each experiment was conducted in triplicate.

### 2.12 BrdU assay

Cells were treated with 10 μM EdU for 2 h, fixed, and permeabilized. EdU incorporation was detected using EdU Cell Proliferation Kit with Alexa Fluor 555(CX003,CellorLab), followed by DAPI counterstaining. The percentage of EdU-positive cells was quantified by fluorescence microscopy or flow cytometry.

### 2.13 Statistical analysis

All data were analyzed using GraphPad Prism 9. For comparisons between two groups, unpaired Student’s t-tests were used. For multiple group comparisons, one-way or two-way ANOVA followed by Tukey’s *post hoc* test was applied. *P* values <0.05 were considered statistically significant. All quantitative data are presented as mean ± standard deviation (SD). Each experiment was independently repeated at least three times (n = 3). Error bars in bar graphs represent SD.

## 3 Results

### 3.1 MK2 is overexpressed in LUAD and is associated with poor prognosis

**FIGURE 1:**
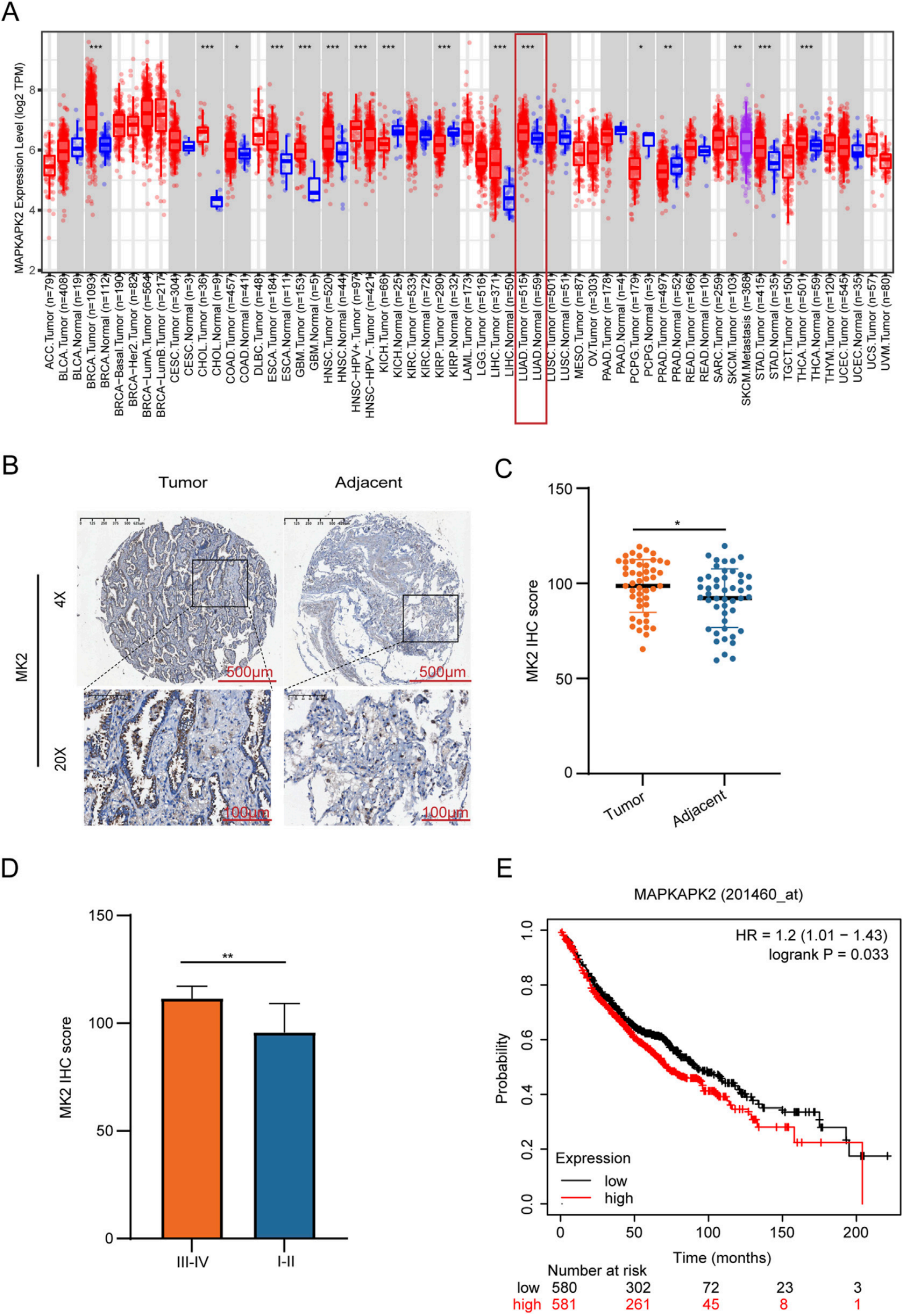
MK2 is Overexpressed in Lung Adenocarcinoma And is Associated With Poor Prognosis. **(A)** Transcriptomic profiling reveals elevated MK2 mRNA levels in lung carcinoma versus normal tissues. **(B)** Immunohistochemistry images comparing MK2 staining in tumor versus adjacent tissues. **(C)** Quantitative histopathology confirms tumor-specific MK2 overexpression via IHC-score quantification. **(D)** Bar graph comparing MK2 IHC scores between different tumor stages, showing higher scores in stages III-IV. **(E)** Kaplan-Meier survival curve indicating the relationship between MAPKAPK2 expression levels and survival probability, with higher expression associated with lower survival. In **(C,D)** panels, unpaired Student’s t-tests were used. In the **(E)** panel, the statistical analysis was performed using the log-rank test. *P < 0.05, **P < 0.01.

Emerging evidence has established MK2 as a commonly dysregulated signaling node in LUAD and multiple human malignancies (Nguyen Ho-Bouldoires et al., 2015; Soni et al., 2019; Jacenik et al., 2023). To systematically characterize its pathobiological relevance, we performed multi-platform bioinformatics validation using the TIMER 2.0 database. Bioinformatics analysis revealed that MK2’s transcription levels are significantly higher in LUAD tissues compared to adjacent non-tumor tissues, suggesting its functional involvement in pulmonary carcinogenesis (Figure 1A). To validate these findings at the protein level, we conducted IHC analysis on 47 matched LUAD-normal tissue pairs using clinically annotated tissue microarrays. Quantitative histoscore analysis confirmed significant elevation of MK2 protein expression in tumor tissues, with representative IHC staining shown in (Figure 1B–C). Further stratification by tumor stage demonstrated markedly higher MK2 expression in advanced-stage (III-IV) LUAD compared to early-stage (I-II) disease (Figure 1D). Clinically, data from the Kaplan-Meier Plotter database indicated a negative correlation between elevated MK2 mRNA levels and overall survival rates in LUAD patients, underscoring MK2’s prognostic significance (Figure 1E).

Collectively, these multi-omics concordant data demonstrate MK2 overexpression at both transcriptional and translational levels in LUAD, mechanistically implicating this kinase in disease progression. The clinical correlation between MK2 overexpression and adverse outcomes warrants functional investigation of its therapeutic targeting potential.

### 3.2 MK2 inhibition decreases the proliferation of LUAD cells

**FIGURE 2:**
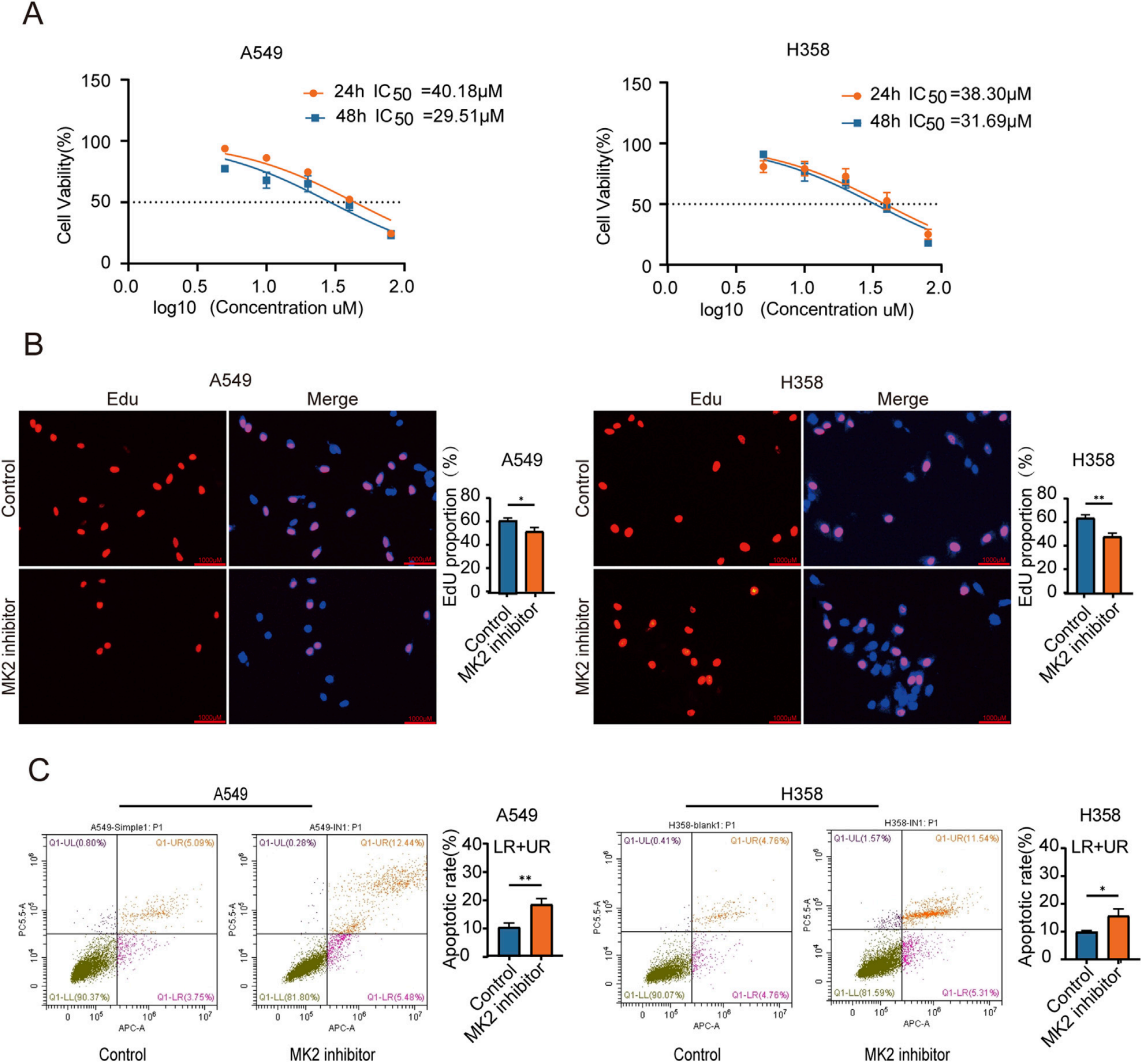
MK2 Inhibition Decreases the Proliferation of LUAD cells. **(A)** The graph shows dose-response curves for A549 and H358 cell lines with IC50 values for 24 and 48 hours. The IC50 value was determined through nonlinear regression(curve fit) analysis of the dose-response data. **(B)** Representative images of EdU assay showing EdU incorporation (red) and nuclei stained with DAPI (blue) in A549 (left) and H358 (right) cells under control and MK2 inhibitor treatment conditions. Quantitative analysis of EdU-positive cells is shown in the corresponding bar graphs (right panels). **(C)** The graph features flow cytometry plots demonstrating apoptotic rates in A549 and H358 cells, with bar graphs comparing conditions. Each experiment was repeated three times (n = 3), *P* values were obtained using Student’s t-test. *P < 0.05, **P < 0.01.

Previous research suggests that MK2-mediated phosphorylation of RIPK1 decreases its affinity for FADD, thereby attenuating TNF-α-induced apoptosis (Jaco et al., 2017; Menon et al., 2017). The therapeutic potential of MK2 inhibition extends beyond direct apoptosis modulation, as it synergistically disrupts oncogenic signaling through dual pathways: sensitizing pancreatic ductal adenocarcinoma (PDAC) to apoptosis via Hsp27 inactivation and restricting breast cancer metastasis by suppressing stromal IL-6 production (Grierson et al., 2021; Murali et al., 2018). To further investigate the role of MK2 in LUAD proliferation, we established *in vitro* models using A549 and H358 cell lines. MK2 expression or activity was inhibited using both siRNA and an MK2 inhibitor (Huang et al., 2011). Comparative analysis of genetic (siRNA) *versus* pharmacological (MK2-IN-1) MK2 suppression revealed comparable efficacy in modulating proliferation and invasion (Supplementary Material 1A-E), prompting subsequent focus on MK2-IN-1 for target specificity validation and translational relevance. Preliminary determination of IC50 values for the MK2 inhibitor (MK2-IN-1) revealed that the IC50 for A549 and H358 were 40.18 µM and 38.30µM, respectively, at 24 h, decreasing to 29.51µM and 31.69  μM at 48 h (Figure 2A). Crucially, 20 μM MK2-IN-1 exhibited no significant cytotoxicity in non-transformed BEAS-2B lung cells (Supplementary Material 1F), underscoring its tumor-selective targeting potential. Functional characterization through EdU incorporation assays revealed marked reduction in LUAD proliferative capacity following MK2 inhibition (Figure 2B), paralleled by a 2fold increase in apoptosis rates as quantified by Annexin V/PI dual staining (Figure 2C). These findings collectively establish that MK2 inhibition disrupts malignant homeostasis by concurrently suppressing proliferation and activating apoptosis in LUAD models, with tumor cell-selective efficacy highlighting its therapeutic promise. These findings show that MK2 inhibitors significantly inhibit the proliferation of LUAD cells, suggesting that MK2 may play an important role in LUAD cell proliferation.

### 3.3 Inhibiting the activity of MK2 reduces the EMT of LUAD cells

**FIGURE 3:**
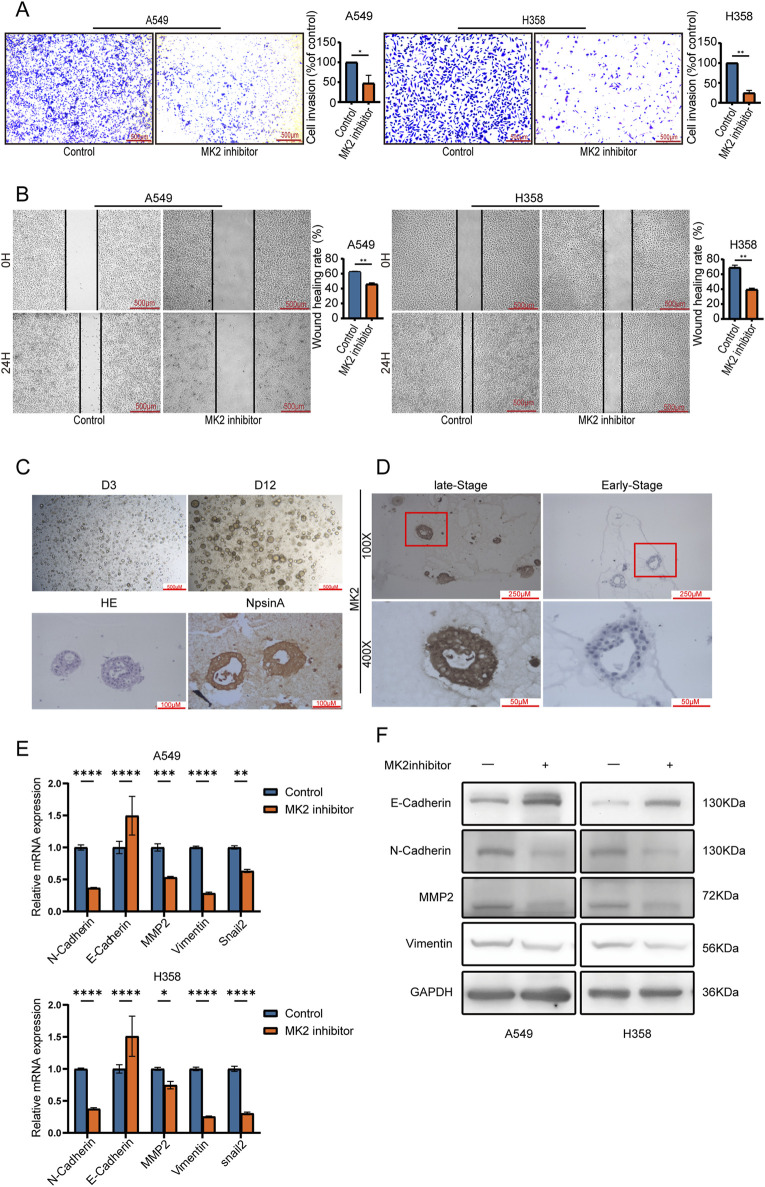
Inhibiting the activity of MK2 reduces the EMT of LUAD cells. **(A)** Transwell invasion assay showing reduced invasion of A549 and H358 cells treated with MK2 inhibitor compared to control groups. Quantification of cell invasion is presented as a percentage of the control group. **(B)** panel B displays wound healing assays at zero and twenty-four hours with bar graphs illustrating healing rates. **(C)** Verification of patient-derived lung cancer organoids (PDLCOs). Dynamic culture: D3 and D12 images showed progressive 3D growth. H&E staining revealed tissue architecture; Napsin A staining confirmed adenocarcinoma origin. **(D)** IHC staining for MK2 in LUAD organoids derived from early-stage specimens (surgical samples) and late-stage specimens (malignant pleural effusion). **(E)** Relative mRNA expression levels of EMT markers were assessed in A549 and H358 cells treated with MK2 inhibitor compared to control groups. **(F)** Protein levels of molecular markers of EMT were examined using Western blot after MK2 inhibition. Each experiment was repeated three times (n = 3). *P* values were obtained using Student’s t test. *P < 0.05, **P < 0.01, ***P < 0.001, ****P < 0.0001.

Given the established correlation between MK2 overexpression and the metastatic potential in various cancers, including melanoma (Wenzina et al., 2020), gastric cancer (Qeadan et al., 2020), colorectal cancer (Ray et al., 2018), breast cancer (Murali et al., 2018) and other tumors; this study investigates MK2-driven invasion-metastasis cascades in LUAD. Functional validation using a homologous LUAD cell model (A549 and H358 cells)showed that the use of MK2 inhibitors greatly reduced tumor cell invasiveness in the Transwell Matrigel assay (Figure 3A). Consistent with these findings, wound healing assays showed a significant inhibition of migratory capacity following inhibition of MK2 viability, and quantitative analysis confirmed a time-dependent inhibition of wound closure (Figure 3B). Next, we further validated the correlation between MK2 and lung adenocarcinoma metastasis at the organoid level. We collected early surgical specimens and advanced malignant pleural effusions for patient-derived carcinoid (PDLCOs) cultures, and verified the biological properties of PDLCOs by a multidimensional technique, where lung adenocarcinoma carcinoids showed cell clustering at the early stage of culture (D3), and in mature carcinoids (D12), the carcinoids were observed to show a dense three-dimensional structure. Subsequently, the lung adenocarcinoma cell origin was further verified by HE staining and the molecular marker for lung adenocarcinoma, Napsin (Figure 3C). Patient-derived organoids from early-stage surgical specimens and advanced malignant pleural effusions demonstrated progression-dependent MK2 upregulation, with advanced-stage organoids exhibiting higher MK2 expression via immunohistochemical quantification (Figure 3D).

EMT stands as a fundamental process in normal embryonic development and serves as a prevalent factor initiating tumor invasion and metastasis (Christofori, 2003; Wheelock and Johnson, 2003; Hazan et al., 2004). As previously documented, EMT is intricately linked with the metastatic progression of various cancers, encompassing liver, ovarian, pancreatic, and breast cancer alike (DiMeo et al., 2009; Cano et al., 2010; Ponnusamy et al., 2010; Xu et al., 2017). To investigate MK2’s role in LUAD-associated EMT, we analyzed transcriptional and translational dynamics of EMT markers following MK2 inhibition. qRT-PCR profiling revealed coordinated transcriptional reprogramming, with significant upregulation of epithelial marker E-cadherin and marked downregulation of mesenchymal regulators N-cadherin, vimentin, MMP2, and Snail following inhibition of MK2 activity (Figure 3E). Consistent with transcriptional changes, Western blot analysis demonstrated protein-level restoration of epithelial phenotypes, evidenced by enhanced E-cadherin expression and reduced mesenchymal marker abundance (Figure 3F; Supplementary Material 2A-D).

These multi-omics findings establish MK2 as a pivotal regulator of EMT plasticity in LUAD. The conserved capacity of MK2 to orchestrate EMT-driven malignancy across LUAD positions it as a therapeutic target for disrupting metastasis-initiating pathways.

### 3.4 MK2 can modulate the AKT/MYC signaling pathway

Previous studies have uncovered that MK2 plays a promoting role in the progression of nasopharyngeal carcinoma by activating the AKT/MYC signaling pathway, which is known to be a key driver in tumorigenesis (Deng et al., 2018). Specifically, the activation of this pathway by MK2 has been associated with enhanced cellular processes that facilitate tumor growth and progression. Additionally, accumulating evidence has emphasized the critical involvement of the AKT/MYC signaling pathway in promoting tumor invasion and metastasis (Wei et al., 2019; Su et al., 2024; Liang et al., 2020), two hallmark features of aggressive cancers. These findings underscore the importance of understanding how MK2 interacts with this pathway to regulate these malignant behaviors.

**FIGURE 4:**
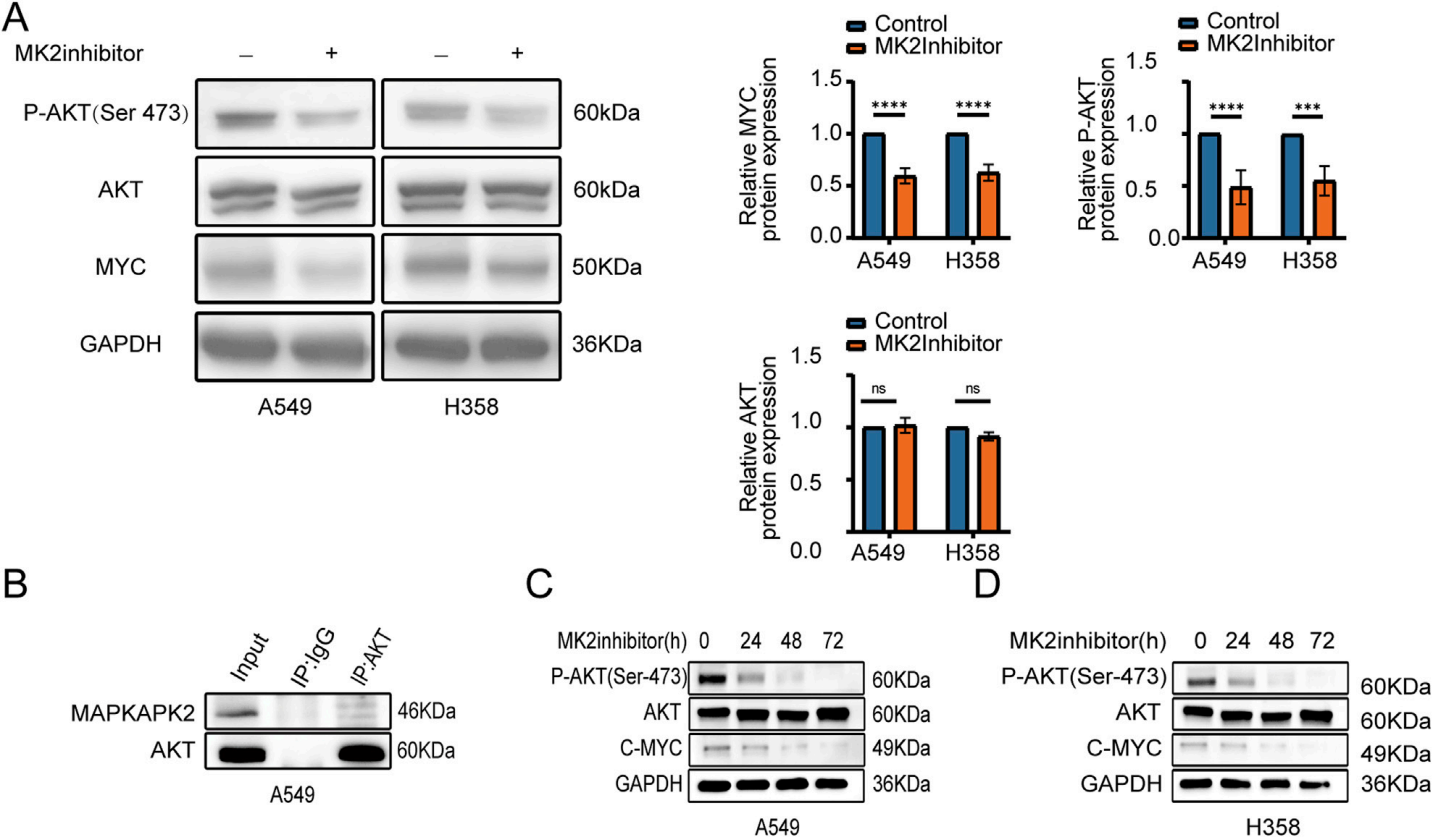
MK2 can modulate the AKT/MYC signaling pathway. **(A)** AKT, P‐AKT(Ser473), and C-MYC expression after MK2 inhibitor treatment in LUAD cells (A549, H358), with GAPDH as loading control. **(B)** Co-IP in A549 confirms MK2-AKT interaction. **(C–D)** Time course (0–72h) shows sustained decrease in P-AKT and C‐MYC after MK2 inhibition in A549 **(C)** and H358 **(D)**, indicating prolonged AKT/MYC pathway suppression. Each experiment was repeated three times (n = 3). *P* values were obtained using Student’s t-test. *P < 0.05, **P < 0.01, ***P < 0.001, ****P < 0.0001.

Given the central role of the AKT/MYC pathway in cancer metastasis, we sought to further elucidate the regulatory effects of MK2 on this signaling cascade. Our experimental data demonstrated that the suppression of MK2 activity led to a marked decrease in the levels of phosphorylated AKT (p-AKT Ser-473) and c-MYC proteins in both A549 and H358 cell lines. This suggests that MK2 modulates the pathway primarily through influencing the phosphorylation state of AKT and the stability or expression of c-MYC. Notably, total AKT protein abundance remained unaltered, pinpointing MK2’s modulatory role in AKT activation status rather than proteostatic regulation (Figure 4A). To confirm a mechanistic interaction, we performed co-immunoprecipitation (Co-IP) assays in A549 cells. The results revealed a direct interaction between MK2 and AKT (Figure 4B), supporting MK2’s involvement in regulating AKT phosphorylation status within this signaling axis. The signaling activation observed over a short period of time (e.g., 24 h) may not adequately represent the persistence of the signaling pathway, so we again extended our analysis to 48 h and 72 h of MK2 inhibitor treatment (Figures 4C–D), and we observed that, in both cell lines, the reduction of p-AKT and MYC was most pronounced at 48 h and 72 h, which confirms that MK2 activity Inhibition of MK2 activity leads to sustained inhibition of AKT phosphorylation and MYC expression over time, implying that MK2 does not only activate the AKT/MYC signaling pathway initially, but that it may also play an important role in maintaining the persistence and strength of these signals. These findings collectively establish MK2 as a kinase-dependent gatekeeper of AKT/MYC-driven oncogenic signaling, coupling post-translational modification to transcriptional reprogramming in metastatic progression.

### 3.5 MK2 regulated the EMT of LUAD cells by the AKT/MYC signaling pathway

**FIGURE 5:**
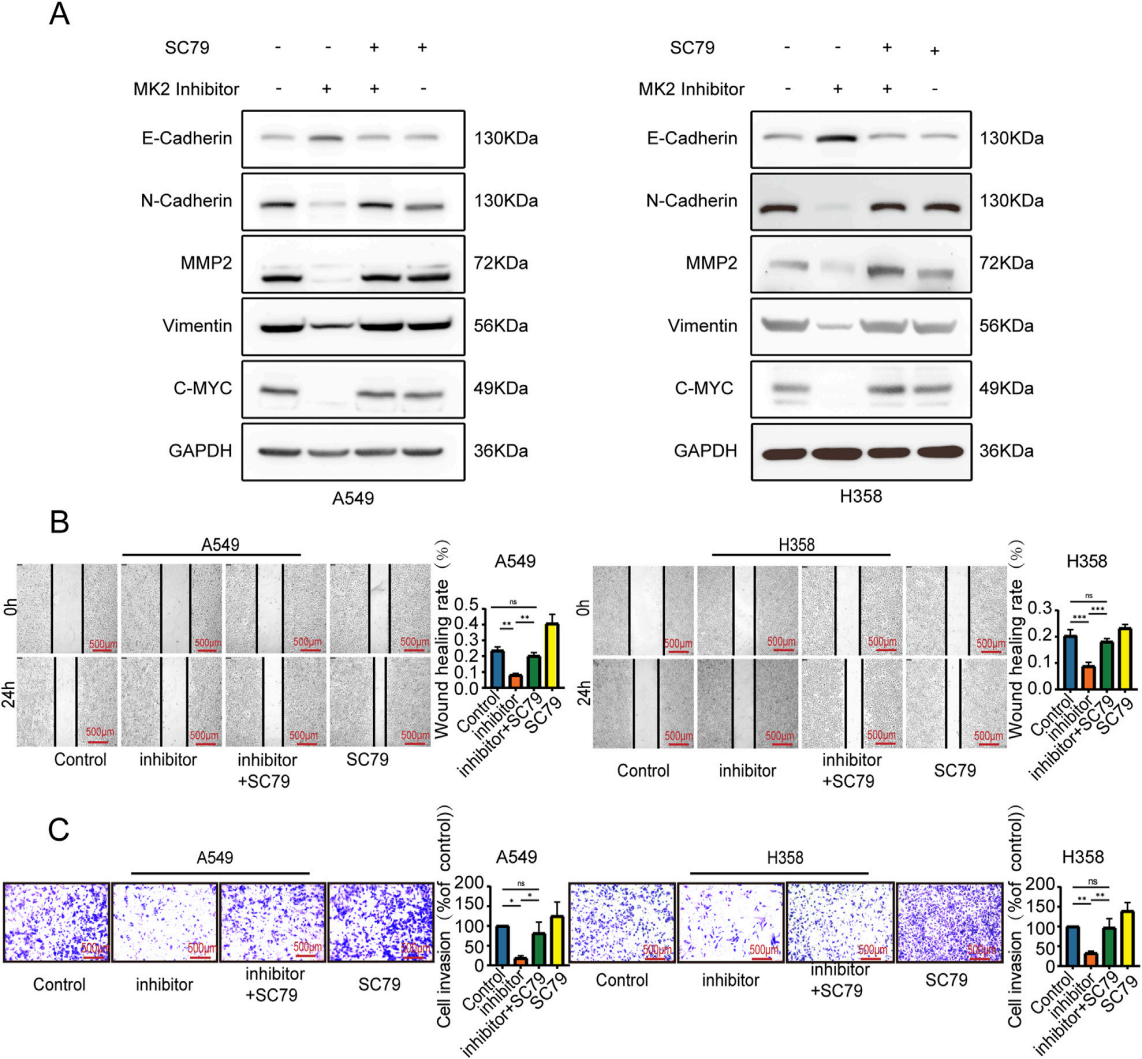
MK2 regulated the EMT of LUAD cells by the AKT/MYC signaling pathway. P**(A)** Western blot detected EMT markers and MYC expression. **(B)** Wound healing assay measured migration (24h post-scratch). **(C)** Transwell assay evaluated invasion. Groups: MK2 inhibitor (MK2-IN-1, 20μM, 24h); MK2 inhibitor + SC79 (MK2-IN-1 pretreatment 6h, then SC79 20μM); SC79 alone (20μM, 24h). Each experiment was repeated three times (n = 3). *P* values were obtained using Two-way ANOVA. *P < 0.05, **P < 0.01, ***P < 0.001, ****P < 0.0001.

**FIGURE 6:**
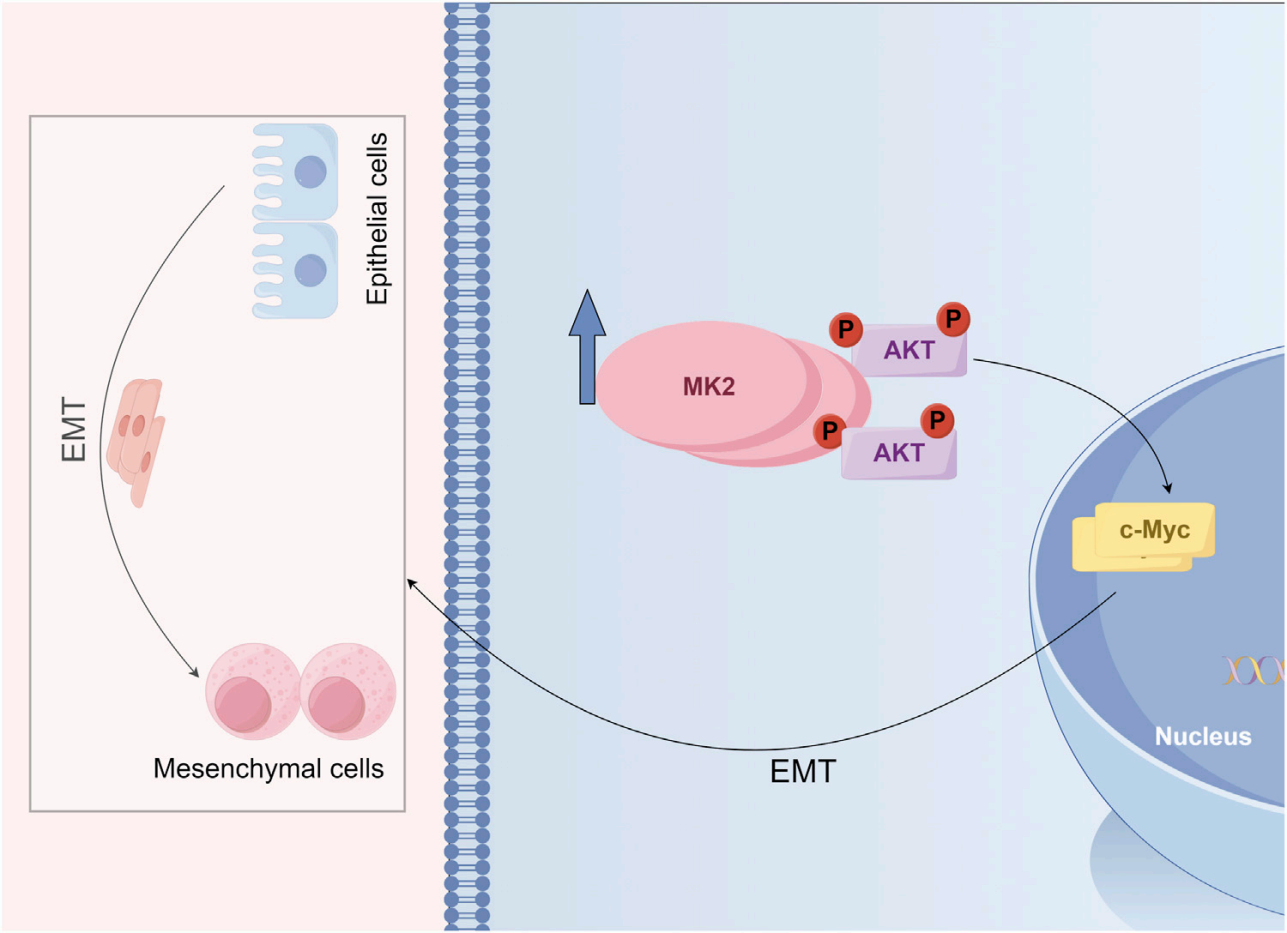
Schematic model illustrating the role of MK2 in regulating the epithelial-mesenchymal transition (EMT) and AKT signaling.

To further ascertain whether the anti-invasive and EMT-modulating effects of MK2 inhibitors in LUAD are mediated through the AKT/MYC pathway, we employed SC79, an activator of the AKT/MYC pathway. Notably, co-administration of SC79 with MK2 inhibitors completely abrogated the inhibitor-induced suppression of MYC and mesenchymal markers (vimentin, MMP2, N-cadherin), effectively reinstating their baseline expression profiles (Figure 5A). Functional complementation assays further demonstrated that AKT/MYC pathway activation rescued the impaired metastatic potential of LUAD cells, as evidenced by restored migratory and invasive capacities in MK2 inhibitor-treated populations (Figures 5B–C). Taken together, our results confirm that MK2 interacts with AKT, promoting AKT phosphorylation and, in turn, enhancing C-MYC expression. The dose-responsive reversibility of MK2 inhibitor effects through pathway activation definitively positions this signaling axis as the mechanistic linchpin connecting MK2 to LUAD progression (Figure 6). These findings establish the AKT/MYC pathway as the dominant downstream effector mediating MK2’s pro-metastatic functions in LUAD, bridging kinase activity to EMT-driven metastatic reprogramming.

## 4 Discussion

In LUAD, MK2 activation is regulated by cellular stress and inflammatory responses. Cellular stress (such as oxidative stress) and inflammatory cytokines (like TNF-alpha and IL-1beta) activate the p38 MAPK pathway, inducing MK2 phosphorylation and regulating tumor cell proliferation and migration (Xu et al., 2025). Furthermore, Bag-1 promotes MK2 activation through the Raf-1-dependent MAPK pathway, while NHERF1 regulates oxidative stress responses by recruiting MK2 in liver cancer cells (Hayashi, Salzet). These upstream signaling pathways play a critical role in MK2 activation and contribute to the regulation of tumor cell behavior, driving LUAD initiation and metastasis.

The p38/MAPK signaling cascade, a ubiquitous signaling enzyme in eukaryotes, plays a multifaceted role in oncogenesis and metastatic dissemination (Sun et al., 2023a). Previous studies have highlighted that the p38/MAPK-specific inhibitor SB203580 can inhibit the proliferation and invasion of breast cancer cells (Liu et al., 2025). Furthermore, research has elucidated that platelet-derived PDGF orchestrates metastatic dissemination in cholangiocarcinoma via p38 MAPK-dependent transcriptional upregulation of MMP2/9 and EMT transcriptional reprogramming (Pan et al., 2020). Despite the pivotal role of p38/MAPK as a therapeutic target, its diverse array of upstream kinases, downstream substrates, and intricate network of regulatory factors contribute to notable side effects (Fiore et al., 2016). Consequently, the identification of novel therapeutic targets within downstream pathway components, with MK2 representing a prioritized candidate, has emerged as a focal point of investigation. In our current study, we identifies MK2, a serine/threonine kinase downstream of p38 MAPK, as a druggable node in LUAD. Immunohistochemical validation revealed tumor-specific MK2 overexpression in LUAD specimens, with functional studies demonstrating that MK2 inhibition suppresses proliferation, migration, and EMT-associated invasion *in vitro*. Crucially, these effects were reversed by AKT/MYC pathway activation, positioning this axis as the dominant downstream mediator of MK2’s oncogenic functions.

MK2 is a serine/threonine kinase positioned downstream of p38 MAPK, pivotal in a myriad of cellular processes including stress response, inflammation, cell proliferation, differentiation, apoptosis, and gene expression regulation (Gujrati et al., 2022; He and Zhao, 2020; Wang et al., 2025; Zhang et al., 2020). Our multi-omics validation in clinical LUAD specimens revealed tumor-specific MK2 overexpression, establishing its pathological relevance. Functional interrogation demonstrated that MK2 suppression not only curbs malignant phenotypes in LUAD models but disrupts the EMT-driven invasion-migration axis, mirroring Henriques et al.‘s observations of MK2-Hsp27-mediated proliferative-migratory circuitry in colorectal carcinogenesis (Henriques et al., 2018). Mechanistically, MK2 inhibition modulates tumor-associated inflammation and mesenchymal transition, exhibiting broad-spectrum anti-neoplastic effects across malignancies (Ray et al., 2018; Murali et al., 2018; Kumar et al., 2009; Ray et al., 2016; Berggren et al., 2019; Morgan et al., 2022). Notably, glioblastoma models exhibit paradoxical MK2 functionality through RSK-EphA2 signaling rewiring, underscoring context-dependent duality in kinase-mediated oncogenic programs (Zhou et al., 2023).

Currently, Numerous recent studies have indicated the involvement of the PI3K/AKT pathway in the metastasis of NSCLC (Zhou et al., 2023), colorectal cancer (Wei et al., 2024) and hepatocellular carcinoma (Chen et al., 2024). Concurrently, the pivotal transcription factor MYC orchestrates the expression of genes crucial for cell growth, survival, and metastasis (Niu et al., 2022; Li et al., 2019; Molteni et al., 2023), underscoring the significance of the AKT/MYC pathway in tumorigenesis. While this pathway’s pathogenic relevance extends to gastric cancer and LUAD (Wei et al., 2019; Su et al., 2024), the upstream regulatory mechanisms governing this oncogenic axis remain incompletely characterized. Hence, we conducted this study to investigate how MK2 regulates the expression of AKT and MYC proteins in LUAD cells, aiming to elucidate its impact on this signaling cascade. Our Western blot analysis revealed that reduction of MK2 activity inhibited AKT phosphorylation and the expression of c-MYC, at the same time, altered the invasion, migration, and EMT profiles of LUAD cells, whereas AKT activation reversed these effects. Obviously, although MK2 can regulate many of signal pathway, the AKT/MYC pathway appears to be a significant downstream pathway regulating EMT in LUAD cells. In hepatocellular carcinoma, treatment with MK2 inhibitor also can block the proliferation and induce the apoptosis via downregulating c-Myc and AKT-1 (Tran et al., 2016). Intriguingly, nicotine-modulated miR-296-3p exemplifies cross-cancer regulation of MK2’s dual signaling outputs, simultaneously targeting both Ras/Braf/Erk/Mek/c-Myc and PI3K/AKT/c-Myc cascades to constrain tumor progression.

Our findings demonstrate that MK2 plays a pivotal role in both the induction and metastasis of LUAD through modulation of the AKT/MYC signaling pathway. This revelation introduces a novel therapeutic target for LUAD treatment, enhances our comprehension of LUAD’s molecular mechanisms, and establishes groundwork for future therapeutic strategies. Notwithstanding these advances, translational considerations warrant deliberate scrutiny. While MK2 inhibition shows promise as a therapeutic strategy, the pleiotropic nature of MK2 in fundamental cellular processes demands careful attention to potential off-target effects. Chronic MK2 suppression may disrupt physiological stress responses and inflammatory signaling cascades, which could lead to unintended adverse effects. As MK2 is involved in multiple cellular pathways, compensation by parallel signaling networks, such as AKT/MYC or PI3K/AKT, may limit the efficacy of MK2-targeted therapies. Therefore, strategies to prevent or overcome compensatory activation of these pathways should be explored, potentially through combination therapies, strategic optimization of dosing regimens and exploration of synthetic lethal combinations with complementary targeted agents emerge as critical priorities to maximize therapeutic efficacy while mitigating on-target toxicities. Secondly, while we delineate AKT/MYC signaling as the dominant effector conduit for MK2-mediated EMT, the precise molecular logic governing MK2-AKT/MYC crosstalk - particularly regarding feedback regulation and intersection with parallel oncogenic networks - remains incompletely resolved. The lack of clarity regarding feedback loops and how MK2 interacts with other oncogenic pathways underlines the importance of future studies to dissect the molecular interactions between MK2 and these pathways to better improve therapeutic strategies. Furthermore, despite the significant findings in our study, we acknowledge certain limitations related to the clinical sample size. The tissue microarray analysis in this study includes 48 paired LUAD samples. While this sample size is common in early-stage studies, it may be relatively modest and not fully representative of the broader patient population. We recommend that future studies include larger cohorts to validate our findings and assess the generalizability of our conclusions across different populations. Additionally, we recognize that the representativeness of the sample set could be influenced by patient demographics, such as age, sex, and ethnicity, as well as disease stages. These factors should be considered when interpreting the clinical relevance of MK2 as a potential therapeutic target. Expanding the study population in future research will be crucial to understand how MK2 expression and its associated signaling pathways might vary in different LUAD subtypes and patient groups.

In summary, while MK2 emerges as a crucial regulator in LUAD metastasis via the AKT/MYC pathway, addressing these limitations through future studies will be critical for validating MK2 as a therapeutic target and optimizing therapeutic strategies for LUAD treatment.

## Data Availability

The original contributions presented in the study are included in the article/Supplementary Material, further inquiries can be directed to the corresponding author.

## Glossary

MK2: Mitogen-Activated Protein Kinase Activated Protein Kinase 2
LUAD: Lung adenocarcinoma
NSCLC: Non small cell lung cancer
MAPK: mitogen activated protein kinase
BLCA: Bladder urothelial carcinoma
BRCA: Breast invasive carcinoma
CESC: Cervical squamous cell carcinoma and endocervical adenocarcinoma
CHOL: Cholangiocarcinoma
COAD: Colon adenocarcinoma
DLBC: Lymphoid neoplasm diffuse large B-cell Lymphoma
ESCA: Esophageal carcinoma
GBM: Glioblastoma multiforme
HNSC: Head and Neck squamous cell carcinoma
KICH: Kidney Chromophobe
KIRC: Kidney renal clear cell carcinoma
KIRP: Kidney renal papillary cell carcinoma
LAML: Acute Myeloid Leukemia
LGG: Brain Lower Grade Glioma
LIHC: Liver hepatocellular carcinoma
LUSC: Lung squamous cell carcinoma
MESO: Mesothelioma
OV: Ovarian serous cystadenocarcinoma
PAAD: Pancreatic adenocarcinoma
PCPG: Pheochromocytoma and Paraganglioma
PRAD: Prostate adenocarcinoma
READ: Rectum adenocarcinoma
SARC: Sarcomav
SKCM: Skin Cutaneous Melanoma
STAD: Stomach adenocarcinoma
TGCT: Testicular Germ Cell Tumors
THCA: Thyroid carcinoma
THYM: Thymoma
UCEC: Uterine Corpus Endometrial Carcinoma
UCS: Uterine Carcinosarcoma
UVM: Uveal Melanoma
PDLCOs: Patient-derived Carcinoid
