# Supplementary material for: MK2-mediated AKT/MYC signaling activation promotes epithelial-mesenchymal transition in lung adenocarcinoma
Source: Front Genet. 2025 Sep 25;16:1615018. doi: 10.3389/fgene.2025.1615018 (PMC12507368; doi:10.3389/fgene.2025.1615018)
Supplement: Supplementary file 1 [file DataSheet1.doc]

**MK2-Mediated AKT/MYC Signaling Activation Promotes Epithelial-Mesenchymal Transition in Lung Adenocarcinoma Supplementary files**

**Supplementary Materials and Methods**

**Patient-Derived LUAD Organoid Culture**

a. Solid Tumor Specimen Processing

Fresh tumor tissues obtained from LUAD surgical resection or needle biopsy were transferred to 50mL conical tubes and rinsed 2-3 times with cold advanced DMEM/F12 containing penicillin/streptomycin on a shaker at 200 rpm, 4°C for 5 minutes. After washing, tissues were minced into fine fragments or paste-like suspensions using sterile ophthalmic scissors in 1.5 mL tubes. The homogenized tissue was transferred to 15 mL tubes containing digestion buffer and incubated at 37 °C for 15-30 minutes at 200 rpm, with digestion monitored microscopically every 5 minutes. Once adequate dissociation was achieved, digestion was halted by adding neutralization buffer. The resulting cell suspension was filtered through a 100μm strainer into a 10cm dish, and the filter was washed with cold advanced DMEM/F12. The suspension was centrifuged at 300 g for 5 minutes at 4 °C. If visible red blood cells were present in the pellet, RBC lysis buffer was added and incubated on a shaker at 120 rpm for 5 minutes at 4 °C, followed by centrifugation and two washes with advanced DMEM/F12. The final pellet was resuspended in LUAD organoid culture medium and mixed with ice-cold Matrigel (Corning). Matrigel domes (20 μL each, 10 domes per well) were plated in pre-warmed low-attachment 6-well plates (Corning) and polymerized at 37 °C for 30 minutes. After gelation, 2mL of pre-warmed LUAD organoid culture medium was added to each well and cultures were maintained at 37 °C in 5% CO₂.

**b. Malignant Pleural Effusion (MPE) Processing**

Pleural effusion samples (50-100 mL) were processed within 2 hours of collection. Samples were centrifuged at 3500 rpm for 15-20 minutes, and the cell pellet was filtered through a 100 μm strainer. Red blood cells were removed using lysis buffer as described above, with repeated lysis steps if necessary. For infected or febrile patients, the cell suspension was filtered through a 0.22 μm membrane to reduce contamination risk. Cells were then embedded in Matrigel and cultured following the same procedure as solid tumor-derived organoids.

**Drug Treatment**

MK2-IN-1(Cat.No.HY-12834,MedChemExpress,China), a selective non-ATP-competitive MK2 inhibitor, was dissolved in sterile phosphate-buffered saline (PBS) to prepare a 10 mM stock solution and stored at -20°C. SC79 (Cat. No. HY-18749, MedChemExpress, China), a small-molecule activator of AKT, was dissolved in dimethyl sulfoxide (DMSO) to a stock concentration of 10 mM and stored at −20 °C in aliquots to avoid repeated freeze-thaw cycles. For experimental use, stock solutions were diluted in complete RPMI-1640 medium (Solarbio, China) containing 10% fetal bovine serum (FBS) and 1% penicillin/streptomycin to reach a final working concentration of 20 μM for both compounds. The final concentration of DMSO did not exceed 0.1% in all treatment groups, including vehicle controls. A549 and H358 cells were seeded into appropriate culture vessels (6-well or 12-well plates) at a density of 2 × 10⁵ to 4 × 10⁵ cells/well and allowed to adhere overnight under standard conditions (37 °C, 5% CO₂, humidified incubator). The next day, cells were treated with MK2-IN-1, SC79, or the combination (MK2-IN-1 pretreatment for 6 hours followed by SC79 for an additional 24 hours). For control groups, equivalent volumes of PBS or DMSO were added.

Supplement figure

Supplement figure 1


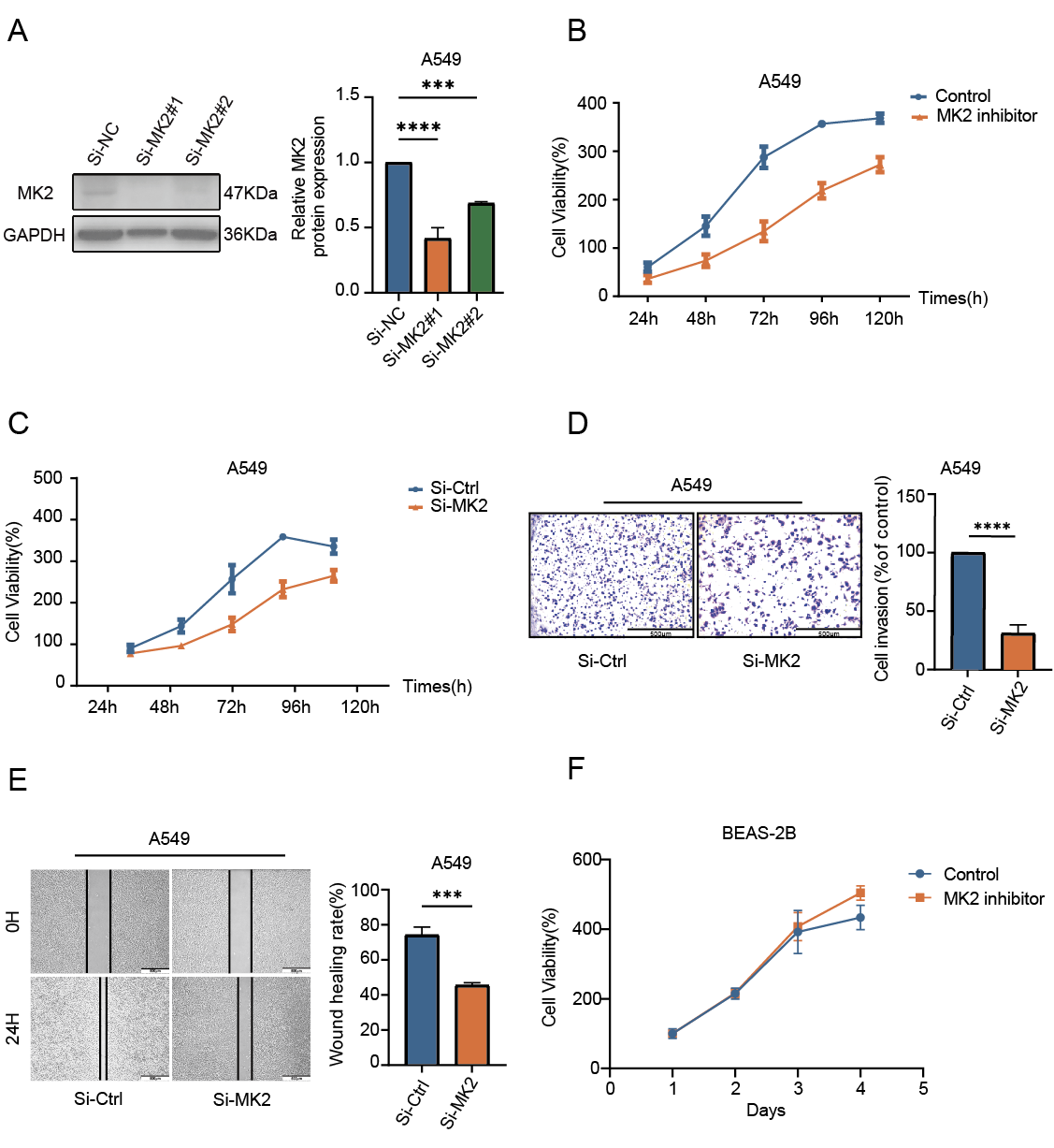


Supplement 1: MK2 Inhibition Decreases the Proliferation of LUAD cells. (A). MK2 protein expression levels after siRNA knockdown in A549 cells. (Left) Representative Western blot showing MK2 and GAPDH protein levels in A549 cells transfected with si-NC (negative control), si-MK2#1, and si-MK2#2. GAPDH was used as a loading control. (Right) Quantification of MK2 protein levels relative to GAPDH, normalized to the si-NC group. (B). The effect of MK2 inhibition or knockdown on the proliferation of A549 cells. A549 cells were treated with an MK2 inhibitor (20µM), and cell viability was measured at 24, 48, 72, 96, and 120 hours using a cell viability assay. (C).The effect of MK2 knockdown on the proliferation of A549 cells. A549 cells were transfected with Si-Ctrl or Si-MK2, and cell viability was similarly measured over 120 hours. (D). The effect of MK2 knockdown on the invasion of A549 cells. Transwell invasion assay showing reduced invasive ability in si-MK2 cells compared to si-Ctrl cells. Quantification of invaded cells is shown as a percentage relative to si-Ctrl . (E).Wound healing assay demonstrating decreased migration in si-MK2 cells at 24 hours compared to si-Ctrl cells. Quantification of the wound healing rate is shown as a percentage. (F). The effect of MK2 inhibitor on the viability of BEAS-2B cells. Cell viability was measured over 4 days in the presence or absence of MK2 inhibitor (20µM). For comparisons between two groups, unpaired Student’s t-tests were used. For multiple group comparisons, one-way or two-way ANOVA followed by Tukey’s post hoc test was applied.* * * P < 0.001, * * * * P < 0.0001.

Supplement figure 2


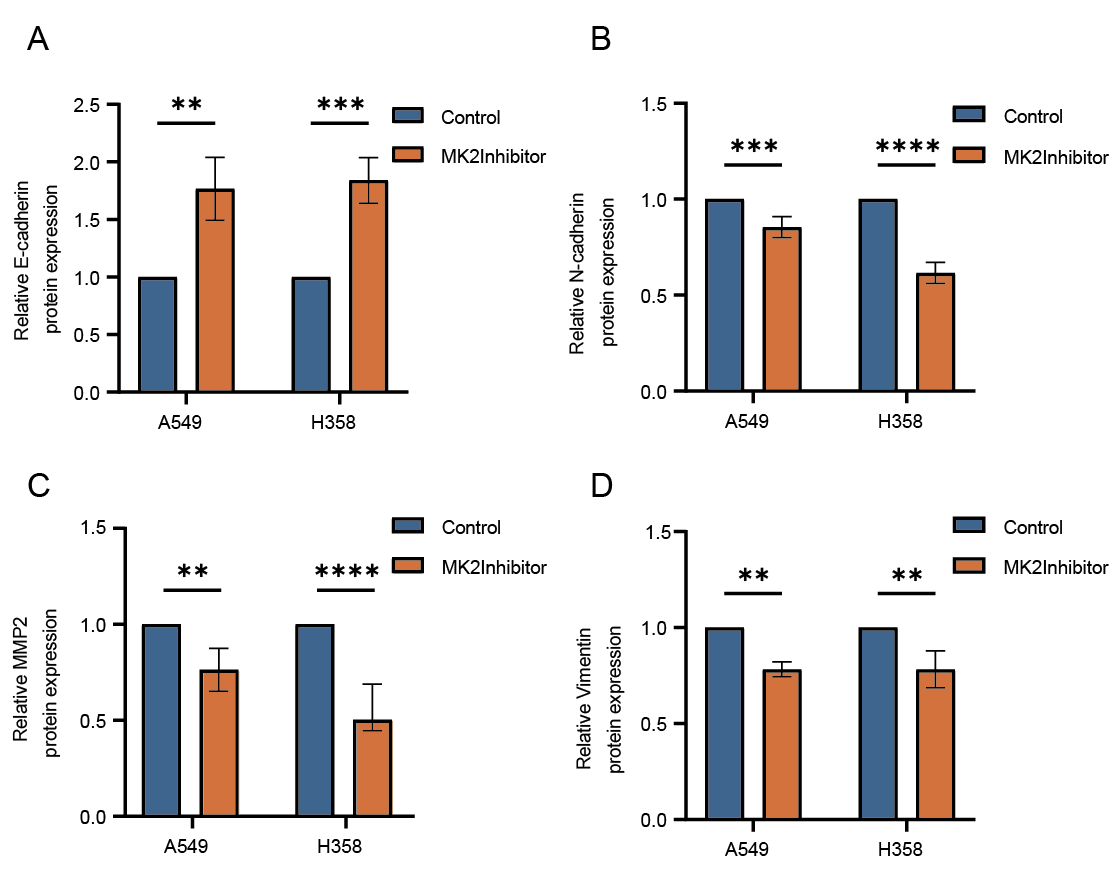


Supplement 2: Relative protein expression of EMT markers in A549 and H358 cell lines after MK2 inhibition. Western blot analysis was performed to determine the protein expression of key EMT markers: (A) E-cadherin, (B) N-cadherin, (C) MMP2, and (D) Vimentin. Relative protein levels were quantified and normalized to the loading control.
